# Template-guided digitally planned patient-specific implant augmentation eminoplasty for chronic recurrent temporomandibular joint dislocation: a prospective study

**DOI:** 10.1186/s40902-026-00508-w

**Published:** 2026-04-29

**Authors:** Mostafa Gafar Ibrahim, Gamal Ali Swaify, Ibrahim Zaitoun, Ahmed Mohamed Medra, Ahmed Yehia Kosba, Mohamed Abdeldayem

**Affiliations:** Faculty of Dentistry, Department of Maxillofacial and Plastic Surgery, Alexandria University, Alexandria, Egypt

## Abstract

**Background:**

Surgical options for chronic recurrent temporomandibular joint (TMJ) dislocation remain heterogeneous. Digitally planned patient-specific onlay augmentation may provide a reproducible mechanical stop while minimizing intraoperative guesswork.

**Methods:**

Eleven patients (> 16 years) with chronic recurrent TMJ dislocation/hypermobility (maximal incisal opening > 40 mm) underwent augmentation eminoplasty using a digitally planned patient-specific implant and CAD/CAM workflow. Clinical outcomes included pain (VAS 0–10) and maximal incisal opening (MIO) assessed preoperatively, at 2 weeks, and at 6 months; radiologic assessment included panoramic TMJ views and MSCT at 6 months.

**Results:**

The cohort was predominantly female (72.7%) with mean age 20.55 ± 4.08 years.

Mean VAS pain decreased from 8.80±0.53 preoperatively to 8.07±0.73 at 2 weeks and 6.29±1.29 at 6 months (all pairwise comparisons p<0.001).

Mouth opening decreased significantly after surgery and remained reduced at follow-up; there was no significant difference between 2-week and 6-month mouth opening (p=0.082).

Two patients (18.2%) developed right-sided postoperative infection requiring implant removal. Clinical signs suggestive of infection were first recognized at approximately 3 weeks postoperatively and ultimately led to implant removal at around 2 months after failure of initial conservative treatment. No clinical recurrence of dislocation/open-lock was documented during the available 6-month follow-up.

**Conclusions:**

Digitally planned patient-specific onlay eminoplasty demonstrated short-term feasibility, reduction in pain, and controlled postoperative mouth opening; however, the observed infection rate and limited functional follow-up warrant cautious interpretation.”

## Introduction

Temporomandibular joint (TMJ) dislocation is a functional derangement in which the mandibular condyle moves out of its normal relationship with the glenoid fossa, most often translating anteriorly beyond the articular eminence. Recurrent episodes can present as frank dislocation requiring assisted reduction or as subluxation that the patient can self-reduce, and both patterns may be associated with pain, joint noises, and progressive functional limitation [1, 2]. 

The pathophysiology of recurrent TMJ dislocation is multifactorial. Contributing factors may include capsular and ligamentous laxity, neuromuscular imbalance with excessive activity of the lateral pterygoid muscle, trauma, internal derangement, degenerative joint change, and behavioral triggers such as yawning or wide mouth opening. Certain neurologic conditions and medication-related dystonic reactions can further predispose susceptible patients to repeated episodes. Because the underlying drivers vary between individuals, outcomes after conservative and surgical interventions are heterogeneous, and recurrence remains a practical challenge [1, 3].

Initial management typically emphasizes conservative measures aimed at reducing spasm, facilitating reduction, and preventing recurrence. These may include activity modification, brief immobilization, physiotherapy, and adjunctive modalities such as injection-based approaches or chemodenervation in selected patients. However, recurrent chronic dislocation that persists despite conservative therapy often requires operative treatment to provide a durable mechanical solution and to reduce the morbidity and anxiety associated with repeated dislocation events [4].

Surgical strategies can be broadly grouped into procedures that remove impediments to condylar translation and procedures that limit excessive anterior translation by creating a mechanical barrier. Eminectomy and related approaches aim to reduce the prominence of the articular eminence to allow free return of the condyle, whereas eminence augmentation techniques seek to reinforce the eminence and restrict forward movement. Although multiple operative techniques have been described, there is no single universally accepted standard; reported outcomes and complication profiles vary, and technical reproducibility can be a limiting factor when implant positioning or bony modification is performed freehand [4–7]. 

Digital planning and patient-specific solutions have emerged as a means to improve reproducibility in cranio-maxillofacial surgery. Patient-specific implants designed from high-resolution computed tomography can conform closely to individual anatomy, potentially improving fit, stability, and load distribution. When combined with CAD/CAM-generated surgical guides, critical steps such as implant seating and drilling trajectories can be standardized, reducing intraoperative variability and shortening the learning curve. In the context of TMJ instability, template-assisted patient-specific eminence augmentation represents a logical evolution of traditional “blocking” procedures, aiming to provide a predictable mechanical stop while minimizing the disadvantages of intraoperative estimation [8–10]. 

The purpose of the present study is to evaluate the clinical and radiologic outcomes of digitally planned, template-guided patient-specific implant augmentation eminoplasty in patients with chronic recurrent TMJ dislocation or hypermobility, with particular attention to postoperative pain, maximal interincisal opening, recurrence control, and procedure-related complications over follow-up.

## Materials and methods

This study evaluated template-guided, digitally planned patient-specific implant augmentation eminoplasty for the management of chronic recurrent temporomandibular joint (TMJ) dislocation and/or TMJ hypermobility. Eleven patients were treated using a standardized workflow integrating virtual planning, CAD/CAM fabrication of patient-specific components, and a consistent surgical approach.

### Participants

Eligible participants were male or female patients older than 16 years with recurrent chronic TMJ dislocation or TMJ hypermobility, defined in this study as a mouth opening greater than 40 mm. Patients were excluded if they had epilepsy, psychological disorders, old TMJ fractures, or TMJ internal derangement.

Patients were considered for surgery after recurrent chronic dislocation/hypermobility causing clinically significant symptoms and failure of conservative management, according to the treating unit’s clinical decision pathway.

## Patient-specific implant workflow: software, materials, and equipment

Virtual planning was performed using Mimics Innovation Suite 19. In the operative workflow (Materialise Co., Leuven, Belgium) to generate a three-dimensional cranio-maxillofacial model. The patient-specific implant material listed for fabrication was ultrahigh molecular weight polyethylene (UHMWPE) sourced from Total Plastics (USA), and the biocompatible resin used for template fabrication was Bio-Compatible Resin from NextDent (Netherlands). The equipment reported for fabrication and quality control included a 3D printer (Phrozen Sonic Mini, Taiwan), a CNC milling machine (China), and a laser scanner (Ceramill Map 400 Scanner, AmannGirrbach GmbH, USA). Fixation hardware was reported as titanium screws (TiGr4ELI, China).

### Preoperative assessment

All patients underwent standardized preoperative assessment including history taking, extraoral facial examination, a full clinical examination to identify any systemic condition that might affect the planned procedure, and intraoral examination. Clinical photographs were obtained. Routine blood investigations were performed. Radiologic evaluation included a standard panoramic radiograph, panoramic TMJ views in open and closed positions (“4 in 1”), and multi-slice computed tomography (MSCT) of the facial bones with three-dimensional reconstruction.

### Virtual planning, fabrication, and verification on a 3D model

CT data with 0.625 mm slice thickness were used for virtual planning. The datasets were imported into Mimics to generate the three-dimensional cranio-maxillofacial model. Patient-specific prostheses were fabricated, and digital templates were manufactured using stereolithography (SLA) printing with resin. The prostheses and templates were trial-fitted on the three-dimensional model to verify passive seating, congruent adaptation to the planned bony contours, and absence of visible rocking before surgery. The guide was designed to reference stable anatomic contours of the zygomatic arch and eminence region in order to standardize implant seating and reproduce the preplanned screw-entry points and trajectories.

### Surgical technique

**Fig. 1:**
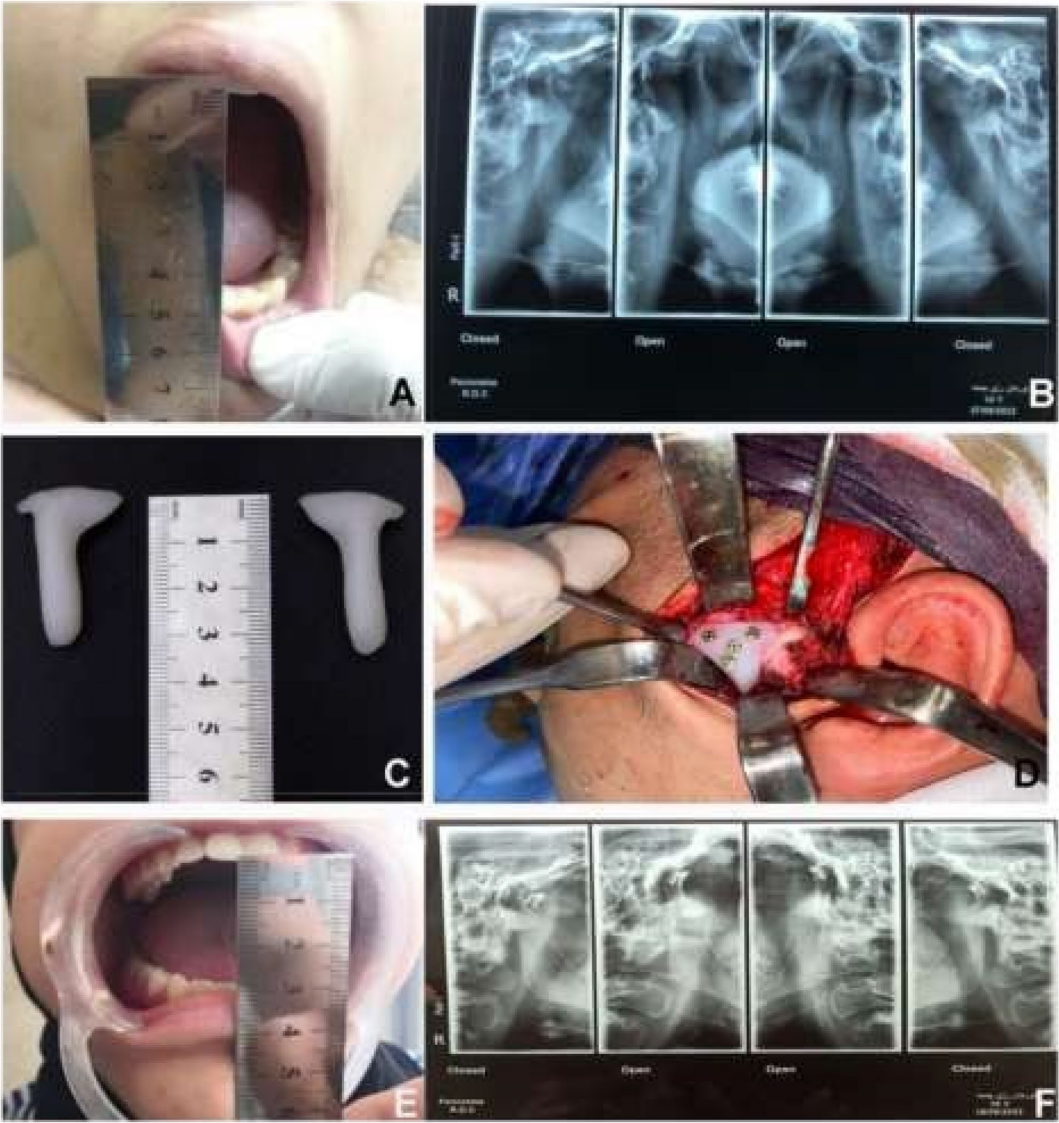
**A** Preoperative MIO about 45mm (**B**) preoperative x ray TMJ open and closed (**C**) custom-made UHMWPE implant (**D**) Intraoperative after placement of the custom-made UHMWPE implant (**E**) Postoperative MIO about 30mm (**F**) Postoperative X-ray TMJ open and closed

All procedures were performed under general anesthesia with nasotracheal intubation. A preauricular incision was used to access the joint and reach the zygomatic arch in the subperiosteal plane. The articular eminence template was placed according to the preoperative design and matched to the zygomatic arch. Four holes were drilled using the template, with two holes positioned superiorly over the arch and two holes positioned inferiorly over the eminence. The template was fixed using four screws, each 7 mm in length. (Fig. 1)

### Postoperative care and clinical outcome assessment

Postoperative management included analgesics, oral antiseptics, and antibiotics according to the institutional postoperative protocol. Patients then proceeded to scheduled clinical follow-up and imaging reassessment.

### Outcomes and outcome assessment

Clinical outcomes included pain and maximal mouth opening. Pain was evaluated using a visual analogue scale (VAS) ranging from 0 (no pain) to 10 (worst pain ever). Mouth opening was evaluated as maximal interincisal opening (MIO) measured in millimeters. In addition to continuous measurement, categorical MIO ranges were documented, including 25–35 mm, 15–25 mm, 5–15 mm, and < 5 mm or nil, translating to grades from 1 to 5 respectively, to support standardized clinical grading.

Clinical recurrence was defined as any postoperative episode of mandibular dislocation or open-lock beyond the articular eminence, whether self-reduced or requiring assisted/manual reduction, as reported by the patient or documented during follow-up.

Outcome assessments were performed at predefined time points. Maximal mouth opening was evaluated preoperatively, intraoperatively, and postoperatively at two weeks and six months. Pain scores were evaluated preoperatively and postoperatively at two weeks and six months. Safety monitoring included documentation of complications, with complication domains explicitly tracked including infection, implant breakage, swelling, hematoma or seroma, persistent pain, and limitation of mouth opening. Patient satisfaction, mastication efficiency, diet tolerance, and dental-care access measures were not prospectively collected. Validated functional outcome instruments such as the Jaw Functional Limitation Scale (JFLS) and Oral Health Impact Profile (OHIP) were not collected in this study.

### Statistical analysis

Statistical analysis was performed using SPSS version 23.0 (SPSS Inc., Chicago, IL, USA). Quantitative variables were summarized as mean with standard deviation and range when parametric, or as median with interquartile range when non-parametric, while qualitative variables were summarized as number and percentage. Normality was assessed using the Kolmogorov–Smirnov and Shapiro–Wilk tests. Given the exploratory nature of this pilot case series and the small sample size, distributional assumptions were assessed using Shapiro–Wilk and Kolmogorov–Smirnov tests. Parametric paired analyses were applied where no major departure from normality was detected; however, the results should be interpreted cautiously given the limited sample size. Comparisons over time were performed using the paired sample t-test for related samples. A 95% confidence interval and a 5% margin of error were specified. Statistical significance was set at *p* < 0.05, with *p* < 0.001 considered highly significant and *p* > 0.05 considered not significant.

## Results

### Participant characteristics

Eleven patients underwent template-guided patient-specific implant augmentation eminoplasty. The cohort was predominantly female, with eight females (72.7%) and three males (27.3%). Age ranged from 17 to 31 years, with a mean age of 20.55 ± 4.08 years; six patients (54.5%) were younger than 20 years.(Tables 1 and 2).

**Table 1:** Sex distribution among study group

Sex	No.	%
Female	8	72.7%
Male	3	27.3%
Total	11	100.0%

**Table 2:** Age "years” distribution among study group

Age "years"	No.	%
<20 years	6	54.5%
≥20 years	5	45.5%
Total	11	100.0%

### Pain outcomes

Pain scores decreased over follow-up for both joints. For the left TMJ, mean VAS pain decreased from 8.91 ± 0.38 preoperatively to 8.23 ± 0.88 at 2 weeks and 6.62 ± 1.14 at 6 months. The reported comparisons showed improvement from baseline to 2 weeks (*p* = 0.020), baseline to 6 months (*p* < 0.001), and from 2 weeks to 6 months (*p* = 0.013). (Table 3)

**Table 3:** Comparison between time intervals according to pain score in left TMJ

Time intervals	Pain Score				Paired Sample t-test		
	Range	Median (IQR)	Mean±SD		MD	t-test	p-value
Pre-operative	8-9.5	9(9-9)	8.91±0.38	P1	0.682	2.776	0.020*
2 weeks post- operative	6.5-9	8(8-9)	8.23±0.88	P2	2.291	7.308	<0.001**
6 months post- operative	5-9	6(6-7)	6.62±1.14	P3	1.610	2.962	0.013*

P1: Comparison between pre-operative and after 2 weeks

P2: Comparison between pre-operative and after 6 months

P3: Comparison between after 2wks. And after 6 months

For the right TMJ, mean VAS pain decreased from 8.68 ± 0.64 preoperatively to 7.91 ± 0.54 at 2 weeks and 5.96 ± 1.41 at 6 months, with all pairwise comparisons reported as *p* < 0.001.(Table 4).

**Table 4:** Comparison between time intervals according to pain score in right TMJ

Time intervals	Pain Score				Paired Sample t-test		
	Range	Median (IQR)	Mean±SD		MD	t-test	p-value
Pre-operative	7-9	9(8.5-9)	8.68±0.64	P1	0.773	4.949	<0.001**
2 weeks post- operative	7-8.5	8(7-9)	7.91±0.54	P2	2.718	9.526	<0.001**
6 Months post- operative	3-7.5	6(5-7)	5.96±1.41	P3	1.950	5.921	<0.001**

P1: Comparison between pre-surgical and after 2 weeks

P2: Comparison between pre- surgical and after 6 months

P3: Comparison between after 2 wks. and post 6 months

When pain scores were summarized as a combined measure across both joints, mean VAS pain decreased from 8.80 ± 0.53 preoperatively to 8.07 ± 0.73 at 2 weeks and 6.29 ± 1.29 at 6 months; all reported pairwise comparisons were *p* < 0.001. (Table 5)

**Table 5:** Comparison between time intervals according to pain score in left and right TMJ

Time intervals	Pain Score				Paired Sample t-test		
	Range	Median (IQR)	Mean±SD		MD	t-test	p-value
Pre-operative	7-9.5	9(9-9)	8.80±0.53	P1	-0.727	5.109	<0.001**
2 weeks post- operative	6.5-9	8(7-9)	8.07±0.73	P2	-2.505	11.812	<0.001**
6 Months post- operative	3-9	6(5-7)	6.29±1.29	P3	-1.777	6.342	<0.001**

P1: Comparison between pre- surgical and after 2 weeks

P2: Comparison between pre- surgical and after 6 months

P3: Comparison between after 2 wks. and post 6 months

### Mouth opening (maximal interincisal opening)

Mean maximal interincisal opening decreased substantially after surgery and remained reduced throughout follow-up. Preoperative mean mouth opening was 45.68 ± 1.78 mm. Intraoperatively, mean mouth opening was 32.55 ± 3.45 mm, decreasing further to 30.64 ± 3.32 mm at 2 weeks and 28.73 ± 3.93 mm at 6 months. The reported analysis indicated that mouth opening decreased significantly from the preoperative value at each postoperative time point. There was no statistically significant difference between the 2-week and 6-month mouth opening values (*p* = 0.082), suggesting relative stability of the postoperative restriction after the early healing period. (Table 6)

**Table 6:** Comparison between time intervals according to Mouth opening (mm)

Time intervals	Mouth opening (mm)		
	Range	Mean±SD	
Preoperative	43-49	45.68±1.78	
Intra-operative	27-38	32.55±3.45	
2 weeks post-operative	25-35	30.64±3.32	
6 Months post-operative	22-35	28.73±3.93	
Paired Sample t-test	MD	t-test	p-value
P1	-13.136	13.809	<0.001**
P2	-15.045	17.563	<0.001**
P3	-16.955	16.823	<0.001**
P4	-1.909	4.605	<0.001**
P5	-3.818	3.231	0.009*
P6	-1.909	1.936	0.082

P1: Preoperative and Intraoperative

P2: Preoperative and 2 week’s post-operative

P3: Preoperative and 6 Months post-operative

P4: Intraoperative and 2 week’s post-operative

P5: Intraoperative and 6 months post-operative

P6: 2 weeks post-operative and 6 Months post-operative

### Complications and reinterventions

Two patients (18.2%) developed clinically diagnosed postoperative infection requiring implant removal. In both cases, the infection involved the right side. Clinical signs were first recognized at approximately 3 weeks postoperatively and included erythema, swelling, and pain over the wound site. Initial conservative management with antibiotics and cold compression led to temporary improvement; however, recurrence of the same symptoms followed by purulent discharge at approximately 2 months postoperatively prompted implant removal. No loose screws were identified at the time of implant removal.

Microbiological cultures were not obtained because purulent discharge became apparent only when implant removal was already indicated. No additional implant-related mechanical complications, including radiologic malposition or fracture, were observed. Following implant removal, no clinical recurrence of dislocation/open-lock was documented during the available follow-up (Table 7).

**Table 7:** Complications distribution among study group

Complications	No.	%
Infection	2	18.2%
No	9	81.8%
Total	11	100.0%

### Radiologic assessment

Postoperative panoramic TMJ imaging in open and closed positions did not show radiologic evidence of implant malposition or persistent dislocation at follow-up. At six months, MSCT assessment demonstrated no osteolysis, malposition, or implant/screw fracture.

## Discussion

### Principal findings

Eminectomy remains one of the most established surgical options for recurrent TMJ dislocation, as it removes the mechanical barrier to condylar repositioning and has demonstrated consistent outcomes across multiple studies [1, 4]. In contrast, augmentation procedures aim to restrict excessive anterior translation by reinforcing the articular eminence.

The present study does not establish superiority of template-guided patient-specific augmentation over eminectomy. Rather, this approach may be considered in selected scenarios, such as patients with pronounced hypermobility requiring mechanical restriction, cases where preservation of native anatomy is desired, or situations in which digital planning may enhance intraoperative reproducibility. Comparative studies are required before its role relative to eminectomy can be clearly defined [11].

### Interpretation of pain improvement

Pain levels showed a notable decrease from baseline to follow-up, with a more significant differentiation observed at the 6-month mark compared to the initial postoperative period. This pattern aligns with clinical expectations: early postoperative pain is often indicative of surgical site inflammation, soft-tissue disruption, and adaptive muscle guarding. In contrast, the later reduction in pain likely reflects a diminishing of repetitive microtrauma associated with recurrent instability events, alongside enhanced neuromuscular confidence during functional activities, such as mouth opening. It’s crucial to recognize that recurrent dislocation is a complex condition with diverse etiological factors; therefore, pain may persist if concurrent issues such as internal derangement, degenerative changes, or myogenous pain mechanisms remain unaddressed, even with successful management of translational movements [1, 12]. Future research would benefit from a standardized approach to determining whether the Visual Analog Scale (VAS) assessments are conducted at rest or during functional activities. Additionally, incorporating validated measures of jaw function is crucial, as pain intensity alone does not adequately reflect the overall benefits observed in temporomandibular joint (TMJ) disorders.

### Mouth opening reduction as a mechanistic endpoint

The observed reduction in maximal interincisal opening reflects the intended mechanical limitation of excessive condylar translation. However, postoperative values in this cohort (~ 28–33 mm) approach the lower boundary of functional adequacy reported in the literature. Comparable augmentation studies have demonstrated postoperative MIO values in a similar range, suggesting that controlled restriction is a common outcome of such procedures [9, 11, 12].

Nevertheless, excessive limitation may impair mastication, oral hygiene, and access for dental care. The absence of validated functional outcome measures in this study prevents definitive assessment of functional acceptability. Future research should define optimal postoperative MIO thresholds that balance joint stability with functional preservation.

A key biomechanical concern with augmentation-based blocking procedures is that normal temporomandibular joint function depends on a coordinated combination of early rotation and subsequent anterior condylar translation. By mechanically restricting translation, eminence augmentation may alter joint kinematics and redistribute loading across the condyle, disc, and glenoid fossa. Although this may reduce instability, it could theoretically increase abnormal contact stresses and contribute to remodeling or degenerative change over time, particularly in patients with pre-existing fossa remodeling, early degenerative disease, or subclinical intra-articular pathology. Because the present study excluded patients with overt internal derangement and did not include long-term surveillance for degenerative progression, the long-term biomechanical consequences of this strategy remain uncertain and warrant dedicated follow-up in future studies.

### Radiologic stability and the value of a patient-specific fit

Postoperative imaging indicated stable positioning of the implants at the six-month mark without any significant failure, reinforcing the notion that anatomically conforming, patient-specific implants can provide more predictable load distribution compared to constructs adapted intraoperatively. This personalized approach is likely to minimize micromotion at the bone–implant interface and enhance seating accuracy, consequently reducing the risk of complications associated with malpositioning. This trend aligns with ongoing advancements in TMJ reconstruction, where the integration of patient-specific CAD/CAM systems and customized prosthetic designs aims to improve fit accuracy and long-term stability in addressing complex TMJ pathologies [13, 14]. Although eminence augmentation differs fundamentally from total joint replacement, both procedures leverage the same core design principle: the implementation of reproducible, anatomy-matched geometries that mitigate intraoperative variability.

### How this approach fits within the existing surgical landscape

The surgical literature addressing chronic recurrent TMJ dislocation encompasses a diverse array of interventions. These include eminectomy for the removal of the articular eminence, various glenotemporal osteotomies with modifications to reposition osseous structures, and mechanical obstruction strategies such as those derived from Dautrey and Leclerc techniques, along with the use of miniplate-based barriers [1, 4, 12, 15–17]. Recent systematic reviews have highlighted significant variability in methodologies, outcome measures, and complication rates among studies, complicating direct comparison and underscoring the necessity for standardized, reproducible approaches and reporting frameworks in this field [4]. In this context, template-guided patient-specific eminence augmentation can be regarded as an advancement of traditional “blocking” techniques. The fundamental objective continues to be the mechanical stabilization of translational movement, but the methodology has evolved to incorporate digital technology, enhancing reproducibility and precision in outcomes.

### Benefits of digital planning and surgical templates

A key benefit of digital planning in maxillofacial surgery lies in the capability to specify implant geometry, seating, and fixation pathways on a three-dimensional model. This approach allows for the intraoperative reproduction of these preoperative decisions via a surgical guide. Both static and dynamic guided techniques have been implemented in various facets of maxillofacial surgery, serving to enhance precision, standardize surgical workflows, and minimize the dependence on intraoperative estimations, particularly in regions with complex anatomical constraints [8, 10]. While guided dental implant surgery differs from eminence augmentation, both share a fundamental principle: template-driven reproducibility. In the realm of TMJ surgery, preliminary clinical studies have investigated the application of template-based access and guided techniques to enhance consistency in minimally invasive TMJ interventions. This approach aims to standardize outcomes and improve precision in surgical practices [18]. The technique used here extends that logic to open surgical eminence augmentation, where millimetric differences in positioning can materially change postoperative range of motion and stability.

### Implant material considerations and clinical implications

The selection of implant material is a critical determinant of both mechanical performance and biological response in augmentation procedures of the temporomandibular joint. In the present study, all patient-specific implants were fabricated from ultrahigh molecular weight polyethylene (UHMWPE), a polymer widely used in orthopedic and craniofacial applications due to its favorable wear characteristics, chemical stability, and biocompatibility.

UHMWPE has demonstrated long-term durability in load-bearing environments, particularly in joint arthroplasty, where its tribological properties contribute to reduced wear and improved implant longevity. In the context of eminence augmentation, these characteristics may support a stable mechanical interface while minimizing frictional interaction with adjacent osseous structures. However, UHMWPE is radiolucent, which may limit direct radiographic visualization and requires indirect assessment of implant positioning and stability [19, 20].

From a biological perspective, polymer-based implants, including UHMWPE, remain susceptible to infection, particularly in anatomically complex and relatively confined surgical fields such as the preauricular region. The infection rate observed in this series (18.2%) underscores that while patient-specific design may improve implant positioning and reduce technical error, it does not eliminate biological risks related to surgical exposure, soft tissue handling, or bacterial contamination [21, 22].

Alternative biomaterials, such as polyether ether ketone (PEEK) and titanium, have also been employed in patient-specific cranio-maxillofacial implants. PEEK offers advantages in radiolucency and modulus of elasticity closer to cortical bone, while titanium provides superior mechanical strength and osseointegration potential. However, each material presents a distinct profile in terms of infection risk, cost, manufacturing complexity, and intraoperative handling [23, 24].

Accordingly, the choice of implant material should not be considered interchangeable or incidental, but rather an integral component of surgical planning. Future comparative studies are warranted to evaluate whether material selection influences complication rates, functional outcomes, and long-term stability in augmentation eminoplasty.

### Complications and risk mitigation

Postoperative infection requiring implant removal occurred in 18.2% of cases, representing a clinically significant complication rate in this small cohort. While patient-specific design may improve positioning accuracy and reduce mechanical complications, it does not mitigate biological risks such as infection, which remain influenced by soft tissue handling, contamination, and host factors [17]. In both infection cases, symptoms were first recognized at approximately 3 weeks postoperatively, initially improved with antibiotics and cold compression, and later recurred with purulent discharge at approximately 2 months, ultimately necessitating explantation. The absence of loose screws at explantation argues against overt mechanical loosening as the primary trigger, although the lack of microbiological data precludes firm conclusions regarding pathogenesis.

Digitally designed, patient-specific implants have the potential to mitigate certain technical complications, such as malpositioning; however, they do not completely eliminate biological risks associated with wound healing, bacterial contamination, soft-tissue management, or individual patient factors.

Importantly, despite implant removal, no recurrence of dislocation was observed during follow-up, suggesting that either early postoperative stabilization or periarticular fibrosis may contribute to maintained joint stability. However, this hypothesis requires further investigation, as longer-term recurrence risk remains unknown.

### Study limitations and future directions

The conclusions that can be drawn are constrained by sample size, lack of a comparator cohort, and relatively short follow-up. Although the approximate timeline, laterality, presenting clinical signs, initial conservative treatment, and indication for explantation could be reconstructed for the 2 infection cases, microbiological cultures and a prospectively standardized infection-management dataset were not available. Accordingly, the causative organisms, depth of infection, and whether these events represented superficial wound infection progressing to deeper implant involvement cannot be definitively established. The exclusion of patients with TMJ internal derangement also limits generalizability, as recurrent dislocation in routine practice may coexist with degenerative or intra-articular pathology, and outcomes in such patients may differ materially.

Outcomes focused primarily on pain and mouth opening, while functional and patient-reported measures were limited; no patient satisfaction, mastication efficiency, diet tolerance, or dental-care access measures were collected, and recurrence should ideally be defined and quantified clinically (episodes, need for manual reduction, time-to-event) rather than inferred mainly from imaging [4], future studies would benefit from a standardized core outcome set that includes pain, MIO, functional limitation scores, recurrence metrics, complication reporting, and radiologic stability. Because this study did not assess operative time, workflow cost, manufacturing expense, or comparative resource utilization, no conclusions can be drawn regarding the cost-effectiveness of patient-specific augmentation relative to established procedures such as eminectomy.

Additionally, because patient-specific onlay eminoplasty protocols have already been described using titanium [9], comparative work examining differences in workflow, material choice, complication rates, and functional trade-offs would be particularly valuable.

## Conclusion

In this prospective case series, template-guided digitally planned patient-specific augmentation eminoplasty using UHMWPE implants demonstrated feasibility and short-term effectiveness in reducing pain and controlling excessive mandibular translation. However, the observed infection rate and reduction in mouth opening highlight important safety and functional considerations.

Given the small sample size, lack of comparator group, short follow-up duration, and absence of validated functional outcome measures, these findings should be interpreted as preliminary. Larger, controlled studies with standardized clinical and functional endpoints are required to define the role of this technique relative to established surgical options.

## Data Availability

The datasets generated and analyzed during the current study are available from the corresponding author upon request.

## Abbreviations

CAD/CAM: Computer—aided design / computer—aided manufacturing
CI: Confidence interval
CT: Computed tomography
DICOM: Digital Imaging and Communications in Medicine
GA: General anesthesia
IQR: Interquartile range
MIO: Maximal interincisal opening
MSCT: Multislice computed tomography
PSI: Patient—specific implant
SD: Standard deviation
SLA: Stereolithography
SPSS: Statistical Package for the Social Sciences
TMJ: Temporomandibular joint
UHMWPE: Ultrahigh molecular weight polyethylene
VAS: Visual analogue scale
